# Pre-sliding of femoral neck system improves fixation stability in pauwels type III femoral neck fracture: a finite element analysis

**DOI:** 10.1186/s12891-023-06631-3

**Published:** 2023-06-21

**Authors:** Yonghan Cha, Jun Young Chung, Chang-Ho Jung, Jin-Woo Kim, Jeyoon Lee, Jun-Il Yoo, Jung-Taek Kim, Yongho Jeon

**Affiliations:** 1 Department of Orthopaedic Surgery, Eulji university hospital, Daejeon, Korea; 2 Department of Orthopedic Surgery, Ajou University School of Medicine, Ajou Medical Center, 164, World cup-ro, Yeongtong-gu, 16499 Suwon, Suwon-si, Gyeonggi-do Korea; 3 Department of Mechanical Engineering, Ajou University, Suwon, Korea; 4 Department of Orthopaedic Surgery, Nowon Eulji Medical Center, Eulji University, Seoul, Korea; 5 Department of Orthopedic Surgery, Inha University Hospital, Incheon, Korea

**Keywords:** Anti-rotation screw, Femoral Neck System, Pauwels type III neck fracture, Pre-sliding technique

## Abstract

**Background:**

Femoral neck fractures are a common injury in older adults and their management presents a significant challenge for orthopedic surgeons. The Femoral Neck System (FNS) was recently introduced for the fixation of femur neck fractures. Although neck shortening was reduced with the FNS, the complication rates were not reduced. Thus, improvements to enhance fixation stability should be made for the FNS. We hypothesized that (1) the pre-sliding technique and (2) the use of longer anti-rotation screw would increase fracture stability. This study aimed to determine the change in fracture stability using the pre-sliding technique and long anti-rotation screw in the FNS for fixation of Pauwels type III femoral neck fractures.

**Methods:**

Finite element models of Pauwels type III femoral neck fracture fixed with pre-sliding FNS and 5-mm longer anti-rotation screw were established. The models were subjected to normal walking load. The material properties of the elements belonging to the bone were mapped by assigning the formulation with the computed tomography Hounsfield unit.

**Results:**

Pauwels type III femoral neck fractures fixed with pre-slided FNS showed better fracture stability, decreasing fracture gap and sliding by 14% and 12%, respectively, under normal walking load. No element of cortical bone in any of the models had an absolute value of principal strain that exceeded 1%. The peak von Mises stress (VMS) of the implants ranged from 260 to 289 MPa, and the highest peak VMS value was 50% lower than the yield strength of the titanium alloy (800 MPa). The longer anti-rotation screw did not affect fracture stability.

**Conclusions:**

The pre-sliding technique using the FNS showed higher fracture stability than the standard fixation technique for a Pauwels type III femoral neck fracture. The longer anti-rotation screw did not contribute significantly to fixation stability. As this finite element analysis considered the inhomogeneous mechanical property of the bone, it offered equivalent mechanical conditions to investigate the components of interest.

## Background

Femoral neck fracture is a common injury, accounting for approximately 50% of all hip fractures [1, 2]. Hip arthroplasty may be considered as a treatment option for femoral neck fractures, considering the patient’s age, fracture displacement, and time interval between trauma and surgery [3–6]. For elderly patients, hip replacement may be considered with significant displacement alone in the treatment of femur neck fractures. However, for younger patients, hip replacement is only considered if there is a combination of significant displacement and risk factors of fixation failure [7, 8]. Internal fixation remains an important treatment option for young patients and non-displaced fractures [9]. Several factors that determine the success of internal fixation include bone mineral density, Pauwels` types, quality of reduction, implant type, and implant positioning. Of these, rigid fixation is one of the controllable factors for a surgeon, and various implants are used to achieve this [10, 11].

**Fig. 1:**
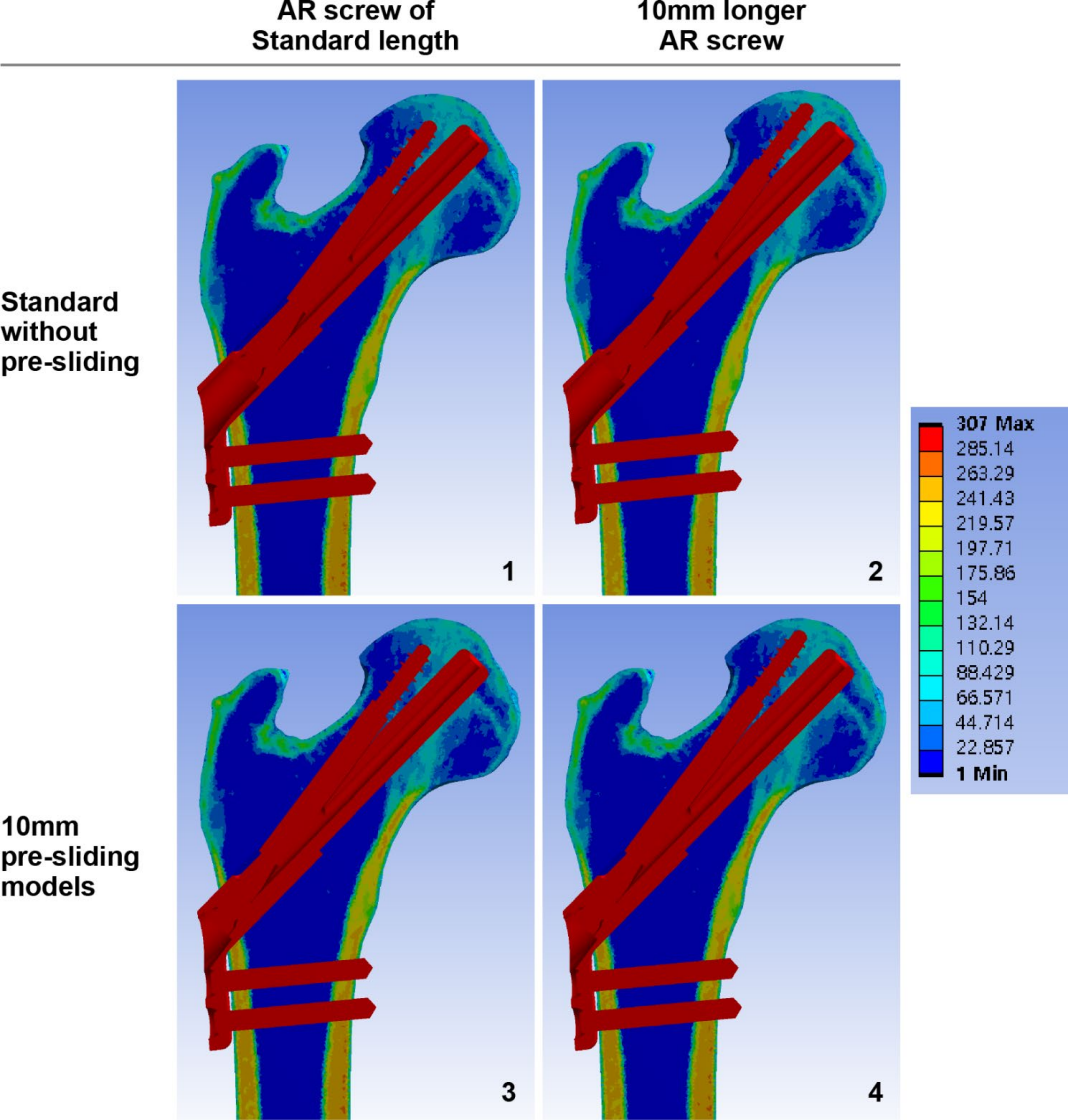
Pauwels type III femoral neck fracture finite element models fixed with the Femoral Neck System. The models were established according to the pre-sliding and long AR screw. Models in the left column have the standard length of AR screws, while those in the right column have one unit longer AR screws. Models in the upper row have AR and bolt assembly in the most protruded position, while the models in the lower row have 10-mm longer bolts at a 10-mm pre-slided position, which makes the bolt’s tip stay the same AR, anti-rotation

**Fig. 2:**
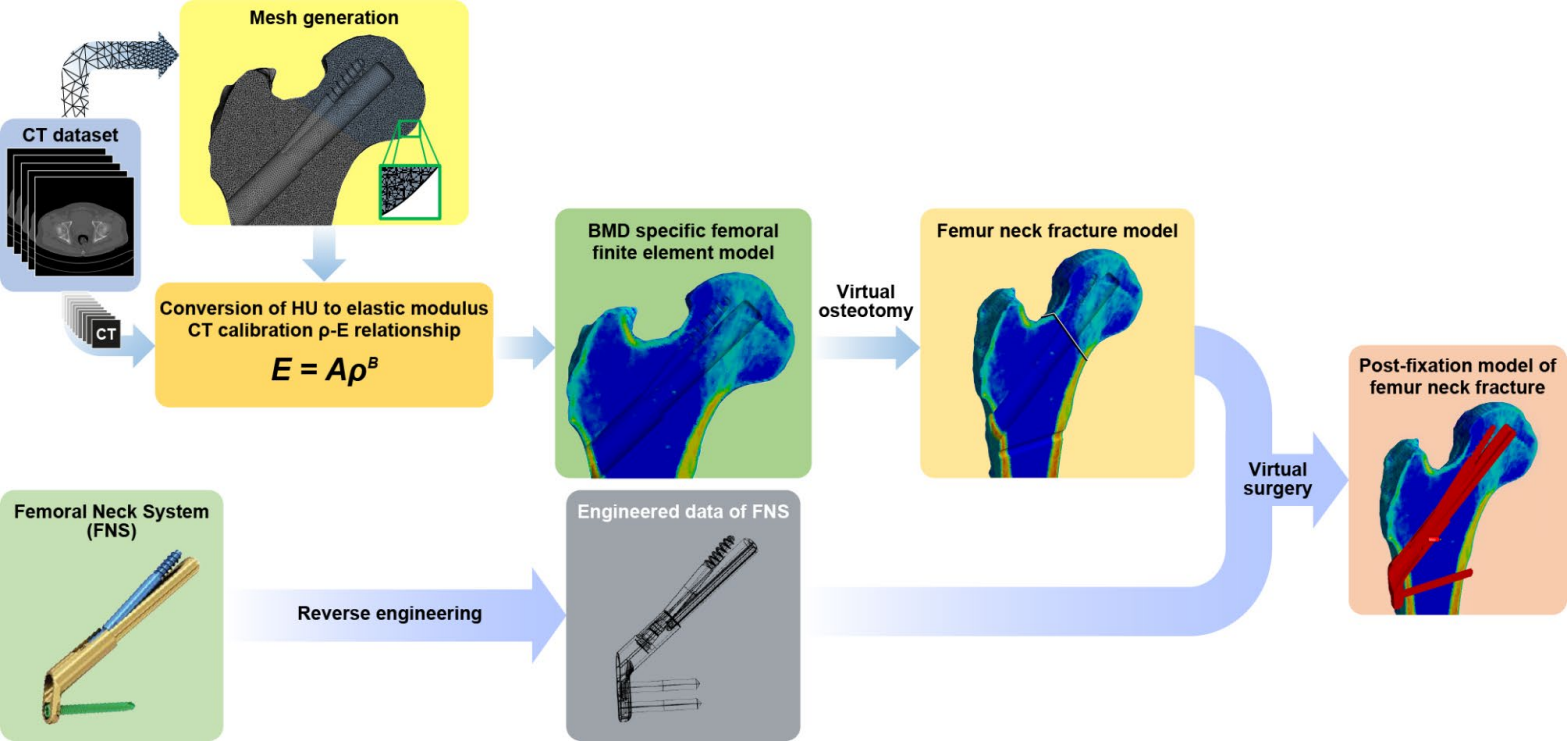
Each femur element was given the material property of the matched voxels of the CT scan by calculating their Hounsfield units CT, computed tomography

The Femoral Neck System (FNS, DePuy Synthes, Oberdorf, Switzerland), for which various clinical results have been recently reported, enables minimally invasive fixation similar to the multiple lag screw technique and has mechanical strength comparable to that of the dynamic hip screw (DHS) [12, 13]. However, disappointing results were reported in a meta-analysis of studies that reported clinical results using a three-triangular multiple lag screw and the FNS for the fixation of femoral neck fractures [14]. Although neck shortening was reduced with the FNS, compared with the three-triangular multiple lag screw, there was no difference in the final functional status and in the rates of complications, such as implant failure and non-union [14]. Thus, we believe that improvements should be made to the FNS to enhance fixation stability.

**Fig. 3:**
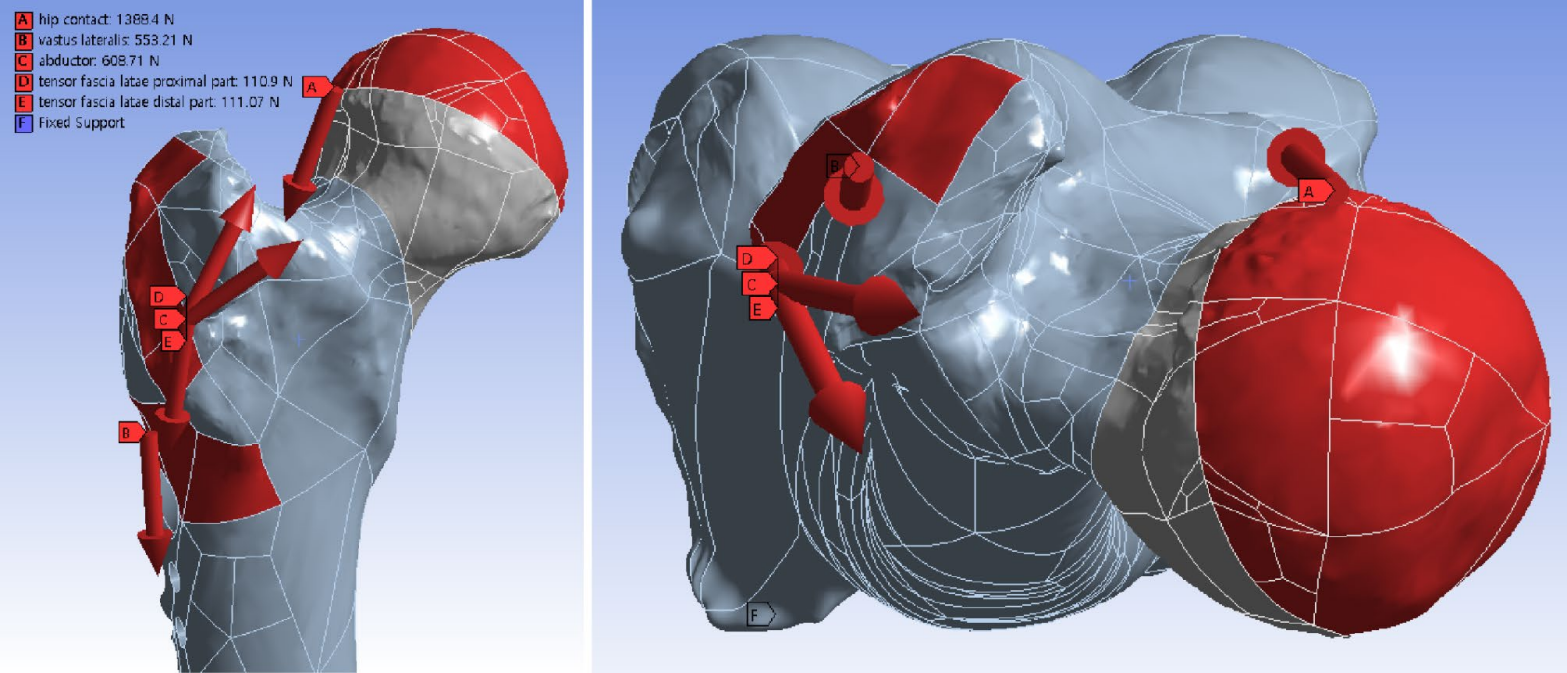
Fracture models virtually loaded in the normal gait condition. Weight load transferred to the hemispheric surface of the femoral head at an inclination of 45° and retroversion of 25° in consideration of the abduction of the acetabulum and the combined anteversion of the acetabulum and femoral neck

Although it has not been clinically proven, two methods have been proposed to increase the stability of FNS fixation [13, 15, 16]. The first is to increase the total length of the implant to be fixed, such as inserting the bolt deeper (pre-sliding technique presented by Cha et al.) or inserting a lateral plate at a distance of approximately 5 mm from the femoral shaft [13, 15]. Second, the FNS should be inserted into the center of the femoral head rather than inferior to the femoral neck anatomical axis [16]. Among these methods, the pre-sliding technique not only allows the bolt of the FNS to be inserted deeper but also positions the anti-rotation (AR) screw further away from the bolt as it passes through the fracture site [15]. Further, inserting an AR screw for as long as possible could increase fixation rigidity. Similarly, in the fixation of multiple screws in femoral neck fractures, proper stability can be obtained when the spacing between screws increases and the screw is inserted deep into the femoral subchondral bone.

**Fig. 4:**
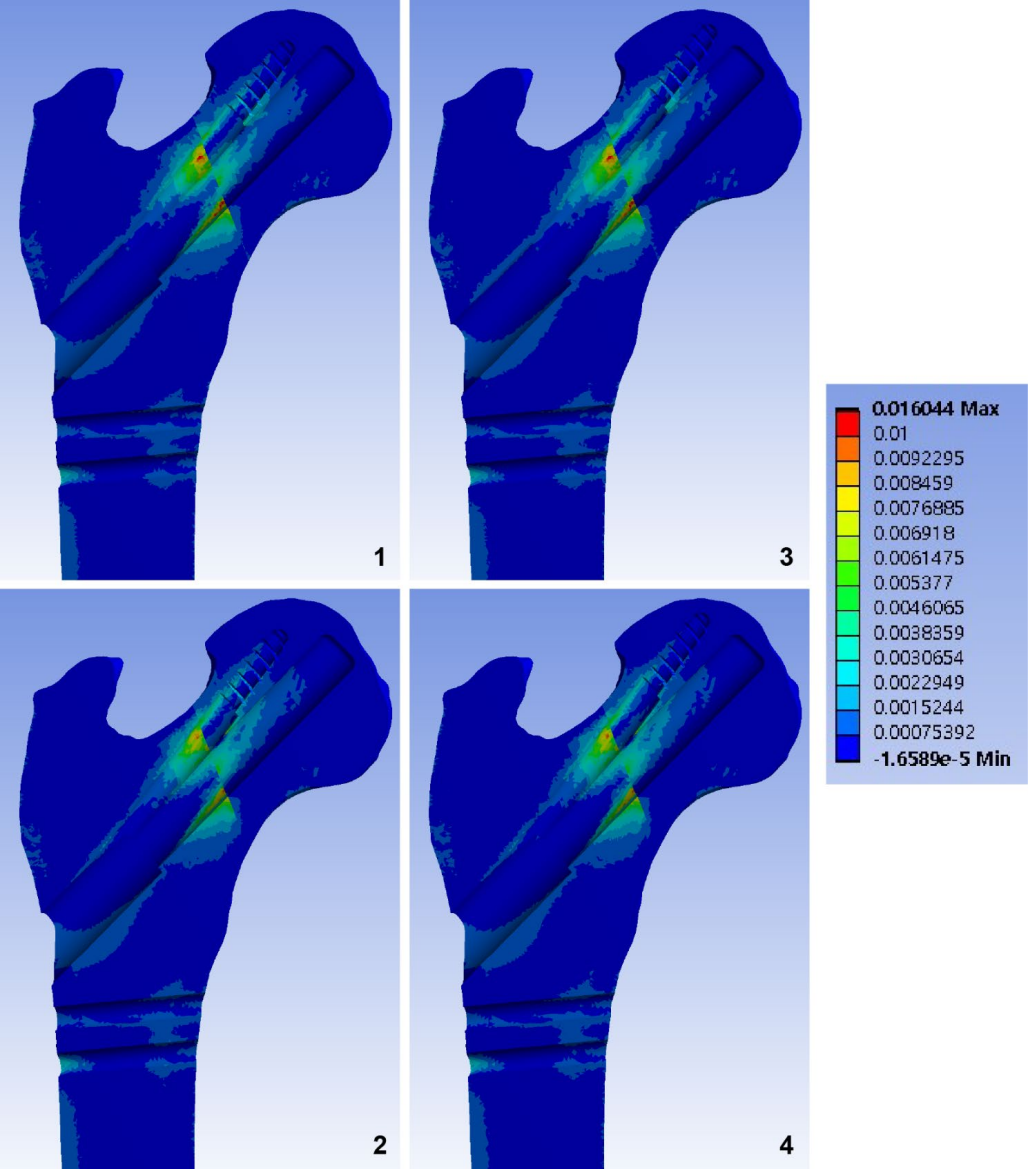
Band graphs depicting the maximum principal strain of the femur. Graphs are arranged in the same sequence as in Fig. 1. All graphs share the color legend

Therefore, we hypothesized that (1) the pre-sliding technique would increase the mechanical strength even if the same length of fixation was obtained compared to fixation without pre-sliding of a femoral neck fracture using the FNS and (2) a longer AR screw would increase fracture stability. The aim of the study was to analyze the mechanical effect on the stability of the fracture site by using the pre-sliding technique and increasing the length of the AR screw in FNS fixation of a femoral neck fracture using finite element models.

**Fig. 5:**
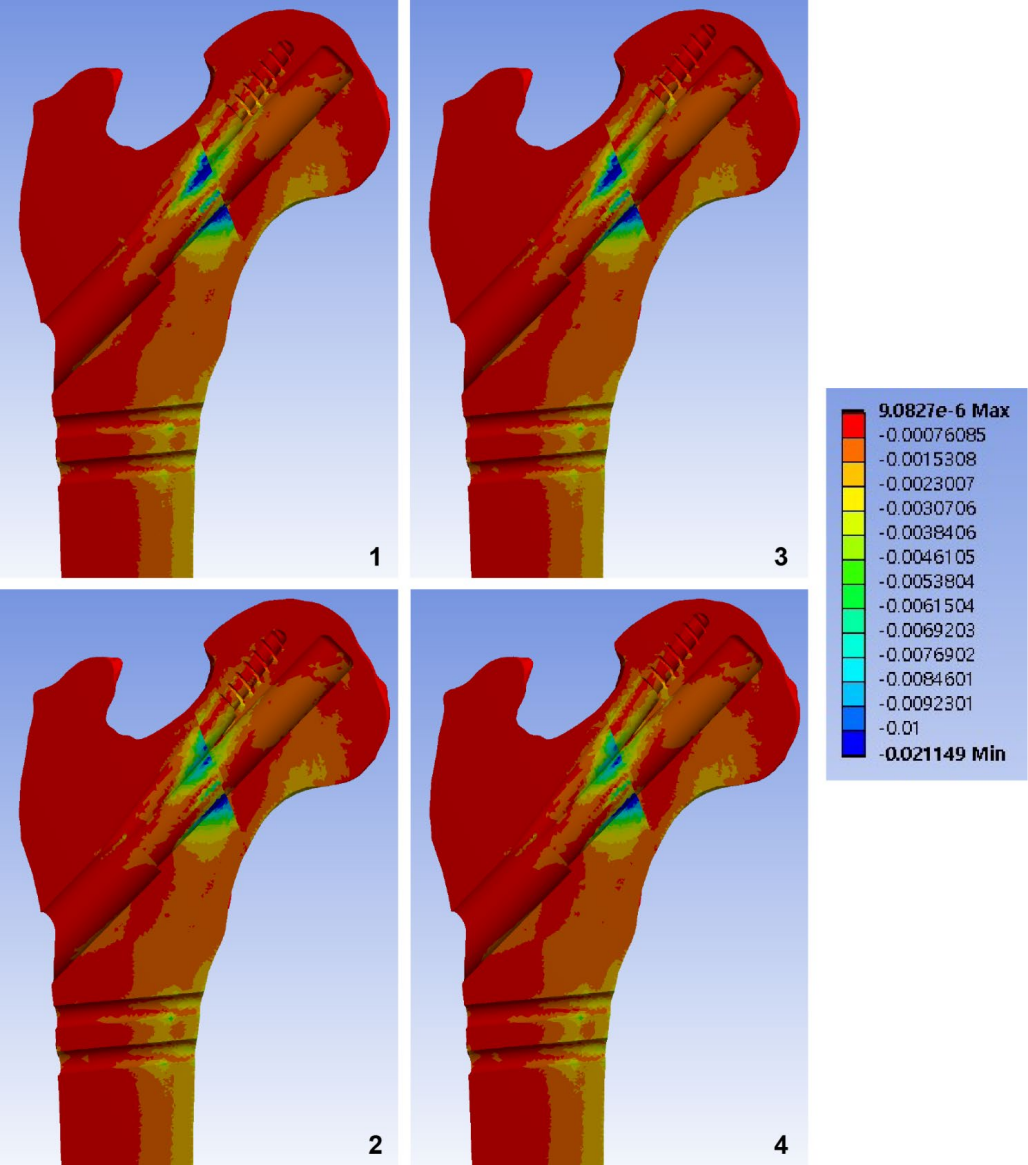
Band graphs depicting the minimum principal strain of the femur. Graphs are arranged in the same sequence as in Fig. 1. All graphs share the color legend

## Methods

Our hospital’s institutional review board (IRB) approved the study protocol. As the computed tomography (CT) scan was part of routine care and the use of the images posed a minimal risk of harm to the patient, the requirement for informed consent was waived (IRB number: AJOUIRB-DB-2022-427).

### Three-dimensional modelling of the femur neck fracture

The finite element model of the osteoporotic femur was reconstructed from the three-dimensional (3D) CT scans of a patient with a hip fracture. Briefly, a pack of femur CT scans was used to evaluate intertrochanteric fractures to establish the finite element model. The male patient’s age, height, and weight were 82 years, 160 cm, and 54 kg, respectively. Although the internal fixation is a primary option for minimally displaced femoral neck fracture even in the elderly patients [7], the mechanical stability after fixation is often compromised in elderly patients due to poor bone density and quality. Materialise Interactive Medical Image Control System Research 22.0 (MIMICS; Materialise, Antwerp, Belgium) software was used to reconstruct the 3D model of the unfractured contralateral femur. By aligning the fracture plane to be inclined by 60° from the horizontal plane using 3-Matic 14 (Materialise), the femoral neck was virtually cut to mimic a model of a Pauwels type III femoral neck fracture [17, 18]. No fracture gap and complete anatomical reduction were assumed.

**Fig. 6:**
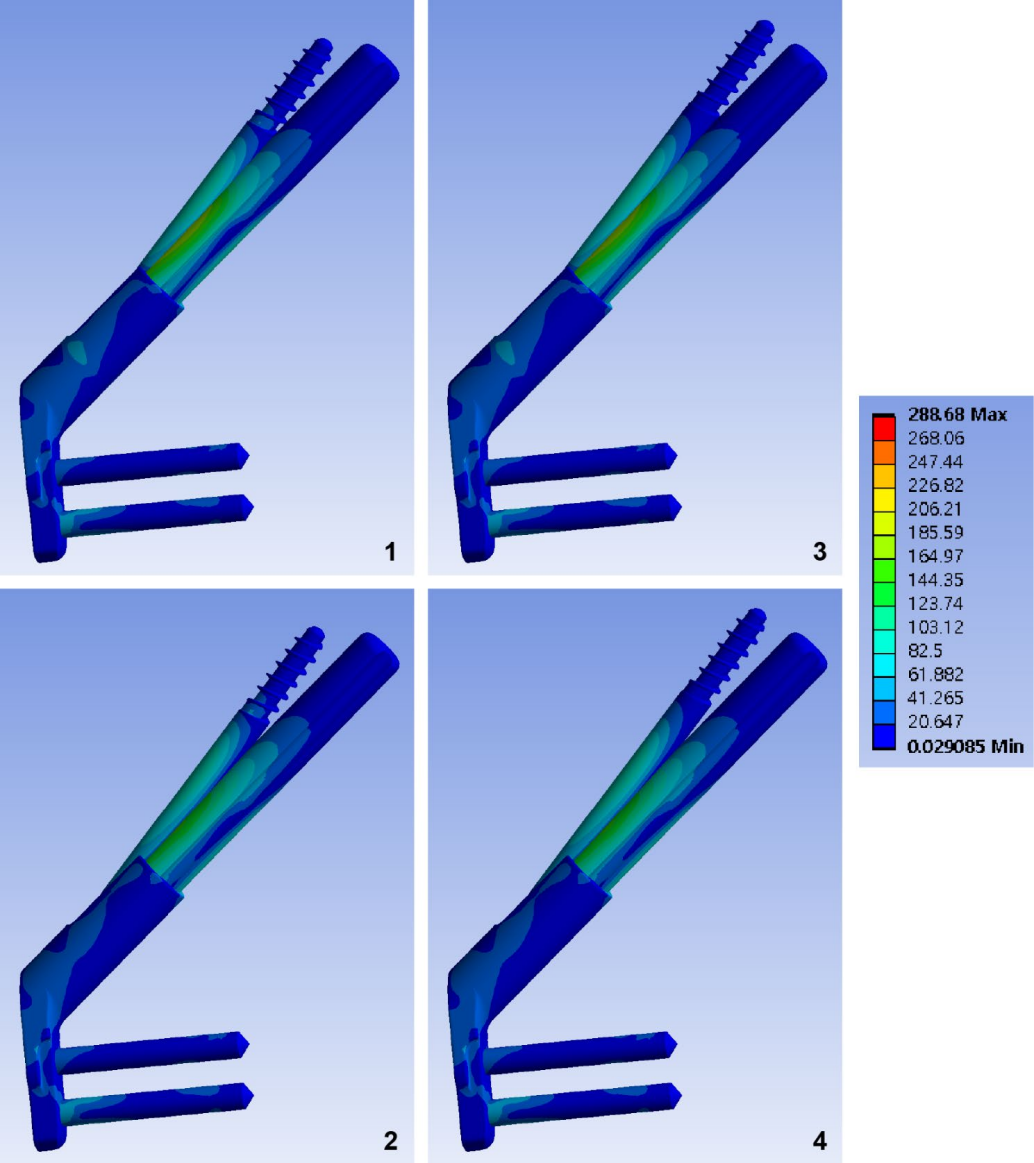
Band graphs depicting von Mises stress of the implant. Graphs are arranged in the same sequence as in Fig. 1. All graphs share the color legend

### Coordinate system

The coordinate system described by Bergmann et al. was used [19]. The best-fitting sphere to the surface of the femoral head was calculated using 3-Matic software. The origin of the coordinate system was located at the center of the sphere. The axis of the femoral diaphysis represented the z-axis, while the frontal plane was defined to include the z-axis and was parallel to the femoral neck axis. The x-axis was assigned to lie in the frontal plane and was normal to the z-axis. The y-axis was normal to both the x- and z-axes.

### Implant positioning

Using 3-Matic software, the fractured femur model was fixed with the 3D FNS model. To establish the standard fixation construct, 95-mm bolts and 95-mm AR screws were virtually assembled in the central trajectory in the neck cortical corridor 5 mm from the bolt tip and the subchondral bone. The bolt was assembled with a two-hole plate in the maximally protruded position. We utilized Boolean subtraction to replicate the bone loss caused by the drilling and reaming procedure of the FNS insertion to reproduce the post-fixation construct [20]. The constructs of the 10-mm pre-slided FNS model were established with 105-mm bolts to match the position of the bolt tip to that of the standard model by using the method of pre-sliding described by Cha et al. (Fig. 1) [15]. As the subchondral bone above the AR screw allows the one-unit longer AR screw in both the standard and 10-mm pre-slided FNS models, additional fixation models with 100-mm AR screw were respectively created from standard and pre-slided models (Fig. 1-1 and 1-3). Overall, four models with variations in pre-sliding and the length of the anti-rotation screw were synthesized.

**Fig. 7:**
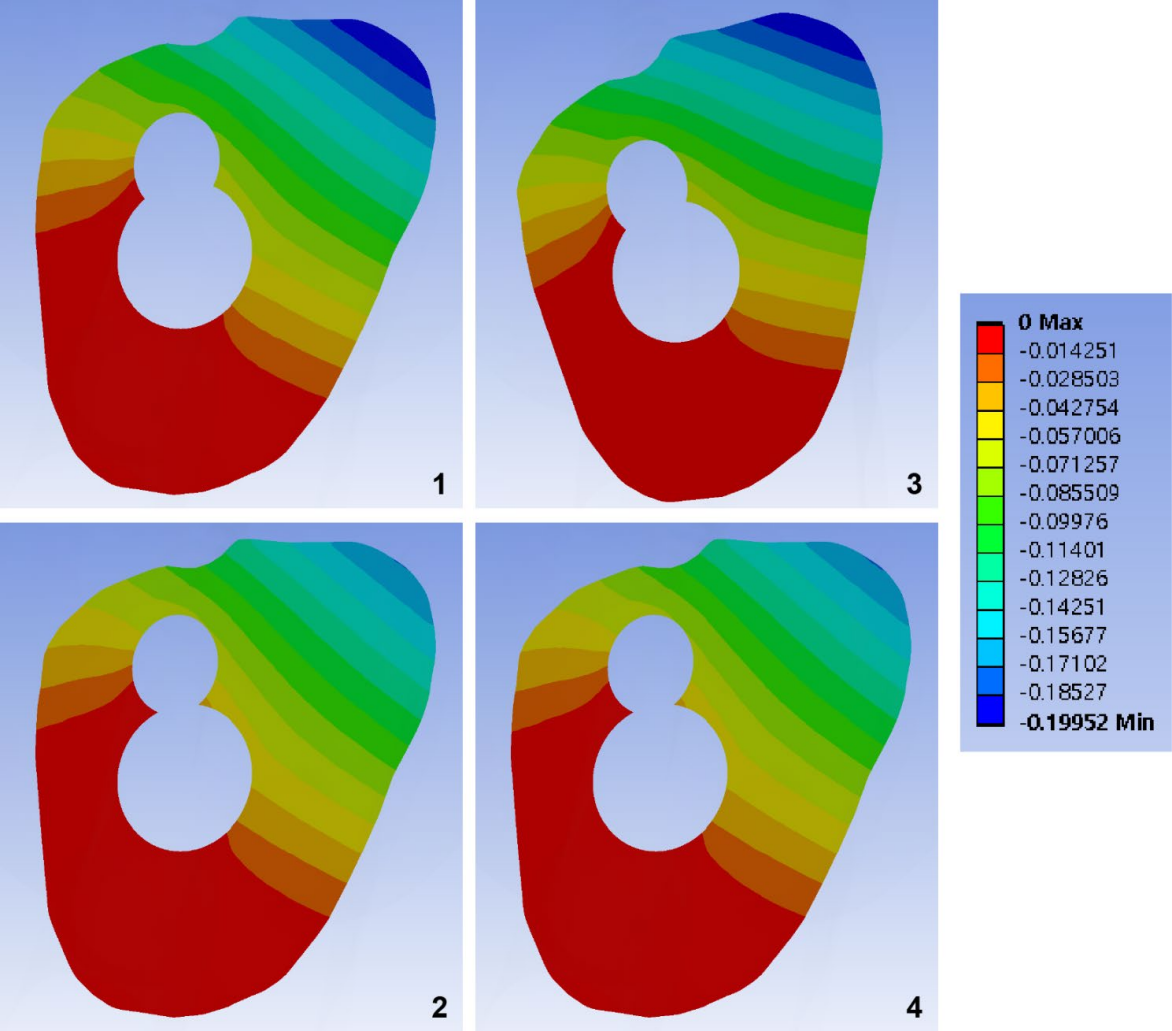
Band graphs depicting gaps between fracture surfaces. Graphs are arranged in the same sequence as in Fig. 1. All graphs share the color legend

### Boundary conditions

The ANSYS 2019 R3 mechanical software (ANSYS Inc., Canonsburg, PA, USA) was used for solving. The interface of the screw joint between the components of metal implants, such as the plate and locking screw head, and the bolt and AR screw were set as bonded. In addition, the interface between the locking screw and the femur shaft was assumed to be bonded. All other interfaces with smooth surfaces without screw joints between the components of the implants and between the implant and bone were assumed to be frictional.

**Fig. 8:**
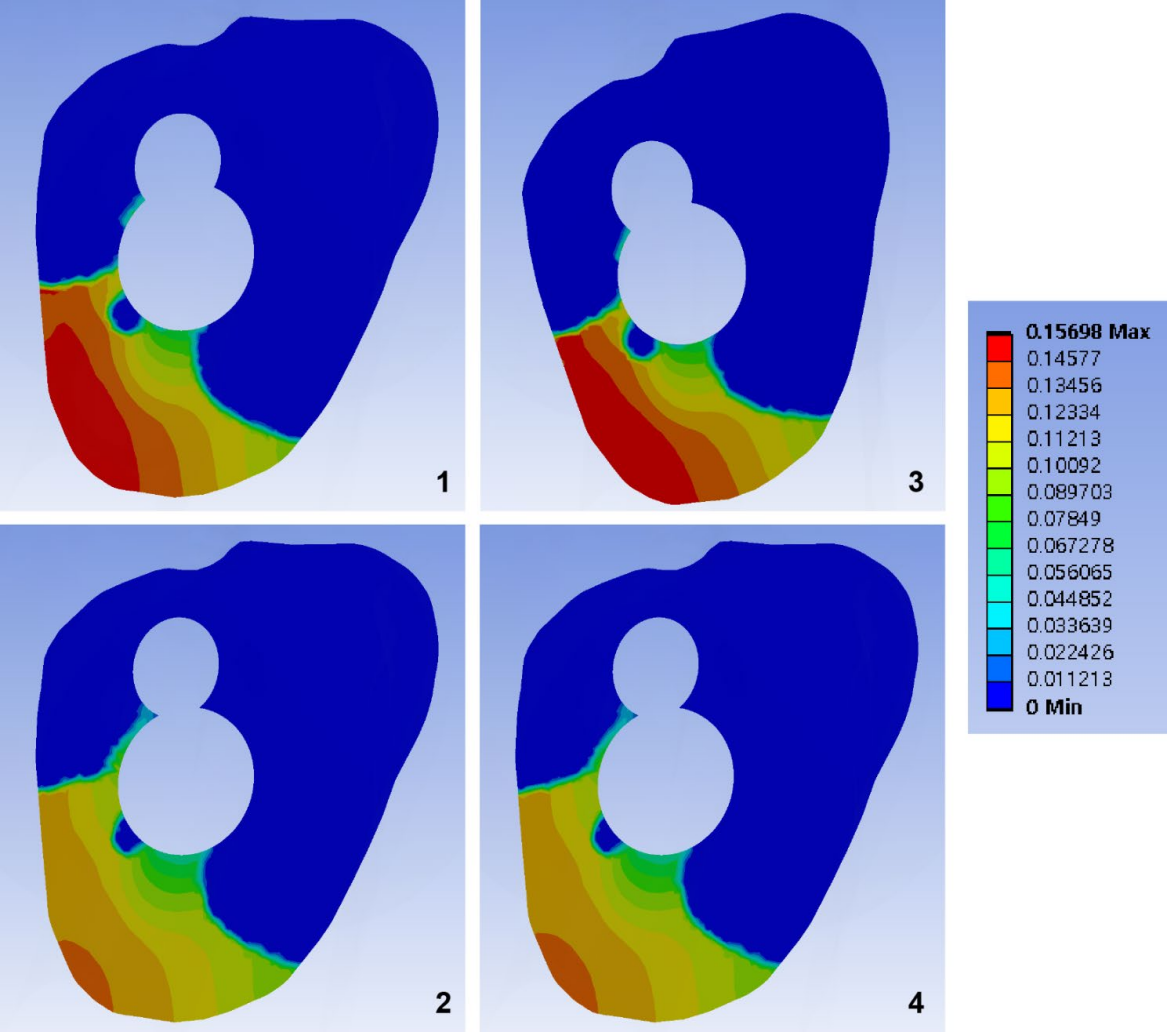
Band graphs depicting the sliding distance between fracture surfaces. Graphs are arranged in the same sequence as in Fig. 1. All graphs share the color legend

The friction coefficients for the bone-bone, bone-implant, and implant-implant interfaces were 0.46, 0.42, and 0.20, respectively [21].

The distal femoral articular face was assumed to be firmly fixed to the Bergmann coordinate system.

### Properties of the materials

The density and Young’s modulus of each bone element were assigned using the mapping method described by Morgan et al. [22, 23], which includes assigning the ash density according to the CT Hounsfield unit. With the ash density, apparent bone density and Young’s modulus can be drawn [23]. The material properties of the bones were assigned into 300 groups (Fig. 2) [24, 25]. Poisson’s ratio of the bone elements was assumed to be 0.3 [22]. The material properties of the titanium alloy (Ti-6Al-7Nb) were assigned to the elements of the metal implants—elastic modulus, Poisson’s ratio, and yield strength, which were 105 GPa, 0.34, and 800 MPa, respectively [26]. The bone and implant were assumed to be isotropic and linearly elastic materials, respectively.

### Loading condition

The load of the normal gait was assumed based on the results of Bergmann et al. and Heller [19, 21, 27, 28]. Simultaneous muscle forces of the hip abductor, the tensor fascia latae, and the vastus lateralis were assigned to the greater trochanter and vastus ridge, respectively (Fig. 3) [21]. A convergence test for the total strain energy was performed.

### Comparative parameters

For the elements that belong to the bone, the maximum and minimum principal strains were evaluated for comparison with the yield strain of the bone, which was set as 1% in absolute value [29]. The von Mises stress (VMS) of the metal implants was evaluated and compared with the yield strength of the titanium alloy, which was 800 MPa [26].

The maximum interfragmentary gap and maximum sliding distance along the fracture plane were assessed to determine mechanical stability at the fracture interface.

We used Microsoft software (Excel and Access) (Microsoft, Redmond, WA, USA) to record parameters. Differences < 5% were considered similar.

## Results

Elements with a peak maximum principal strain over 1% and minimum principal strain under − 1% indicated that the trabecular bone was located in the narrow cleft between the AR screw or under the plate, which supports the bending of the bolt (Figs. 4 and 5).

For the cortical bone, no element of the cortical bone in any of the models had an absolute value of principal strain that exceeded 1%. The cortical elements around the distal screw had a maximum principal strain of 0.5–0.6%, whereas those around the inferior neck had a minimum principal strain of 0.4% (Table 1).

**Table 1:** Interfragmentary motion and stress on the fracture surface and implant with surgical variation in the Femoral Neck System

	95 mm-bolt with 95 mm-AR screw	95 mm-bolt with 100 mm-AR screw	105 mm-bolt with 105 mm-AR screw and pre-sliding 10 mm	105 mm-bolt with 110 mm-AR screw and pre-sliding 10 mm
N. of element	4,818,771	4,819,056	4,828,615	4,827,715
N. of node	6,635,946	6,636,832	6,651,221	6,650,735
Maximum deformation (mm)	13.4	13.4	13.3	13.3
Peak maximum principal strain				
Cortical	0.54%	0.54%	0.57%	0.58%
over 1% volume (ml)	0	0	0	0
Trabecular	1.29%	1.30%	1.60%	1.45%
over 1% volume (ml)	0.44	0.38	0.06	0.11
Peak minimum principal strain				
Cortical	-0.42%	-0.43%	-0.42%	-0.41%
under − 1% volume (ml)	0	0	0	0
Trabecular	-2.10%	-2.11%	-1.87%	-1.85%
under − 1% volume (ml)	36.79	37.23	22.53	24.53
Implant stress (MPa)	261.14	260.06	288.68	287.86
Gap (mm)	0.20	0.20	0.17	0.17
Sliding (mm)	0.16	0.16	0.14	0.14

N: number; AR: anti-rotation

The peak VMS of the implants ranged from 260 MPa to 289 MPa. The highest peak VMS value was 50% lower than the yield strength of the titanium alloy (800 MPa) (Fig. 6; Table 1).

Although the longer AR screw did not provide additional stability at the fracture site compared to the standard length of the AR screw, the pre-sliding of 10 mm decreased the fracture gap and sliding by 14% and 12%, respectively, under a normal walking load (Figs. 7 and 8; Table 1).

## Discussion

In this study, we examined the change in fracture stability using the pre-sliding technique and long anti-rotation screw in the FNS for fixation of Pauwels type III femoral neck fractures. The main results of our study are summarized as follows. First, when performing FNS surgery for femoral neck fracture, using the pre-sliding technique reduced the fracture site gap and sliding by 14% and 12%, respectively, compared to the standard technique. Second, inserting a longer AR screw did not increase the mechanical strength of the fracture site compared to when the standard length of the AR screw was used.

According to the lever balance reconstruction theory, the fulcrum of the lever is located at the center of the hip joint, and the anatomical lever maintains the balance between the lateral tensile stress and the medial compressive stress [16]. If this lever structure is broken by a hip fracture, the patient cannot ambulate. Therefore, surgical treatment aims to stabilize the fracture site through internal fixation and restore the anatomical structure to reconstruct the physiological fulcrum and lever. To achieve this, it is important to control the abnormal tensile and compressive stresses at the fracture site. Wan et al. analyzed the stress occurring at the fracture site in intertrochanteric fracture models with three types of cephalomedullary nails [16]. They reported that the stress applied to the femoral head is transmitted through a blade or screw inserted into the head and that cut-out may occur if stress is concentrated on the implant. Therefore, we believe that stress distribution induced at the fracture site is one of the main factors to consider in treating patients with femoral neck fractures. Further, they reported that, among the three types of nails analyzed in their study, the proximal femur bionic nail (PFBN) had the lowest stress on the screw because it effectively dispersed the tensile and compression stresses on the intertrochanteric fracture surface [16]. We hypothesize that because the two screws of PFBN passed in two directions with a gap at the fracture site, unlike other nails, the fixation area occupied by the implant in the fracture site is widened, which disperses stress and increases the stability of fixation.

In this study, while the bolt tip remained at the same location, the pre-sliding technique of the FNS moved the penetration site of the AR screw on the fracture surface away from that of the bolt. By increasing the distance between the two fixation components, improved fixation stability could be achieved by a longer moment arm against rotational stresses and a longer lever arm against bending stresses. However, the FNS provided a sliding distance of approximately 2 cm when the neck fracture site collapsed. Therefore, a pre-sliding of 10 mm reduced the capacity of fracture sliding. As the collapse of a fracture site greater than 1 cm is reported to be a risk factor for functional disability in femoral neck fractures [30] and for treatment failure, such as nonunion and post-traumatic osteonecrosis of the femoral head [31], limiting the sliding of the FNS could be favorable to fracture healing.

It has been reported that the tip-apex distance (TAD), which represents the insertion depth of the DHS lag screw, affects the failure rate of intertrochanteric fractures. An acceptable TAD was suggested to be 25 mm [32, 33]. In addition, to obtain rigid stability during fixation using three inverted triangular multiple screws in patients with femoral neck fractures, insertion within 3 mm of the femoral head subchondral bone is recommended [34]. The common message from both recommendations is that placing an implant closer to the subchondral bone enhances the chance of successful treatment. However, in our study, compared with the standard length of the AR screw, a 5-mm longer AR screw did not provide additional stability at the fracture site. This might be because the major structure which resists the deforming loads is the bolt of the FNS because it has a larger diameter than the AR screw. Moreover, the long axis of each component might affect the extent of the contribution to stability. Compared to the bolt, the AR screw is supposed to be inserted in the valgus. As the weight load primarily deforms the fracture site in varus, the length of the bolt determines stability instead of the length of the AR screw [13].

There were several limitations in our study. One of these limitations was the use of a single specific model reconstructed from CT scans of an elderly patient, which disregarded the interpatient variations in bone density and geometry. As finite element analysis also makes several assumptions for simplification, there may be doubts concerning whether our findings can be applicable to the general patients in actual clinical environments. Although the femur’s finite element model was created using CT scans of an elderly patient with a Pauwels type III femoral neck fracture, the elements of the femur model were assumed to be isotropic and elastic materials. It may be possible to bridge the gap between simulation and the real-world environment by mapping the material properties based on the grey values of the CT scan. Despite the limitations of simplification, finite element analysis enables the mechanical evaluation of different fixation methods by applying them to identical models, allowing for comparisons between various fixation techniques, as in this study, or providing a useful method for explaining rare but biomechanically caused complications [35]. A loading condition for normal walking was assumed in this study. As the given design of the FNS allocates the bolt and AR screw in a divergent manner, the difference in the length of the AR screw might have exerted rotational stability under the rotational force in the stance or swing phase of normal walking. Further research using various physiological loading conditions could reach a different conclusion from ours. Moreover, our results were not validated by the experiments conducted in this study. Given the realistic reproduction of the fixation construct with reverse engineering of the fixation device and the validated relationship between CT imaging and material properties, our study findings may represent actual clinical solutions.

## Conclusions

The pre-sliding technique using the FNS increases mechanical strength compared to the standard fixation technique for a Pauwels type III femoral neck fracture. Longer AR screws did not contribute significantly to fixation stability. Our study findings may contribute to exploring improved methods of FNS fixation. As the present study was performed *in silico*, further experimental and clinical studies would clarify our findings.

## Data Availability

The datasets used and/or analyzed during the current study are available from the corresponding author on reasonable request.

## Abbreviations

3D: Three-dimensional
AR: Anti-rotation
CT: Computed tomography
DHS: Dynamic hip screw
FNS: Femoral Neck System
IRB: Institutional review board
MIMICS: Materialise Interactive Medical Image Control System
PFBN: Proximal femur bionic nail
TAD: Tip-apex distance
VMS: Von Mises stress
